# Research Progress of Hydrogen Production Technology and Related Catalysts by Electrolysis of Water

**DOI:** 10.3390/molecules28135010

**Published:** 2023-06-26

**Authors:** Haiyao Li, Jun Guo, Zhishan Li, Jinsong Wang

**Affiliations:** 1 Faculty of Metallugical and Energy Engineering, Kunming University of Science and Technology, Kunming 650093, China; hailiyao@outlook.com (H.L.); zhishanli@kust.edu.cn (Z.L.); 2 Faculty of Materials Science and Engineering, Kunming University of Science and Technology, Kunming 650093, China; jswang512@kust.edu.cn

**Keywords:** water electrolysis technique, hydrogen production, alkaline electrolysis, catalyst

## Abstract

As a clean and renewable energy source for sustainable development, hydrogen energy has gained a lot of attention from the general public and researchers. Hydrogen production by electrolysis of water is the most important approach to producing hydrogen, and it is also the main way to realize carbon neutrality. In this paper, the main technologies of hydrogen production by electrolysis of water are discussed in detail; their characteristics, advantages, and disadvantages are analyzed; and the selection criteria and design criteria of catalysts are presented. The catalysts used in various hydrogen production technologies and their characteristics are emphatically expounded, aiming at optimizing the existing catalyst system and developing new high-performance, high-stability, and low-cost catalysts. Finally, the problems and solutions in the practical design of catalysts are discussed and explored.

## 1. Introduction

Hydrogen, as a clean energy carrier, plays an irreplaceable role in the future development of the energy industry. Compared with traditional fossil fuels, such as coal, natural gas, and oil, hydrogen has natural advantages of high calorific value, high energy density, and zero pollution, and is an ideal “clean energy” under the goal of carbon neutrality in China [1,2,3]. Furthermore, hydrogen has a wide range of sources, and water can be used as an important source of hydrogen due to the abundant hydrogen element in water. The calorific value of hydrogen is about three times as high as that of diesel. With the continuous development of hydrogen energy technology and the gradual maturity of hydrogen storage technology, the application of hydrogen energy becomes more and more extensive. Hydrogen energy has been widely used in electric power, heat energy, fuel cells, chemical synthesis, petroleum refining, metallurgical industry, and other fields. Among them, hydrogen energy has a high specific energy in thermal power generation, and the combustion products are pollution-free. Injecting hydrogen into the existing natural gas pipeline for subsequent co-combustion is an efficient hydrogen utilization method, which can make full use of the existing natural gas pipeline network infrastructure and greatly reduce storage and transportation costs. After adding hydrogen, the gas flow in the pipeline is increased, and the hydrocarbon mass ratio of natural gas, the precipitation of carbon particles, and the brightness of flame are decreased. During the combustion process of a hydrogen-doped natural gas boiler, the introduction of hydrogen can inhibit the generation of nitrogen oxides and soot and reduce the emission of CO_2_. Potential applications of hydrogen fuel cells are as follows: (1) They can be used in the automobile field. The product of hydrogen fuel cells is only water. Using hydrogen fuel cells instead of fossil fuel as driving energy for vehicles can effectively reduce environmental pollution. (2) Hydrogen fuel cells can be applied to the field of ships. They play an important role in the composition of new energy structures. Compared with diesel-driven ships, ships with hydrogen fuel cells have longer endurance and meet the auxiliary energy demand of large ships. (3) Hydrogen fuel cells can be used in small unmanned aerial vehicle power systems (UVAs). Compared with the existing UAV battery technology, the endurance of UAVs driven by fuel cells has improved by 4~5 times. In the metallurgical industry, traditional coke steelmaking will produce a large amount of CO_2_. Using hydrogen instead of coke as a reducing agent, the diffusion capacity of hydrogen is higher than that of CO gas obtained by coke oxidation, and the by-product of steelmaking is water, which will significantly reduce carbon emissions and promote the change in the metallurgical industry toward low-carbon and clean production. Additionally, as clean energy with zero carbon emission, hydrogen energy can help people decarbonize, fix carbon emissions, and even achieve negative carbon. Hydrogen energy used as the terminal energy can reduce or even eliminate CO_2_ emissions in the industrial field through the complementary system of hydrogen and electricity.

To satisfy the increasing demand for hydrogen, researchers are constantly exploring existing hydrogen production technologies and developing new hydrogen production technologies. Hydrogen is classified into three categories according to the principle of production mode and different raw materials: chemical reforming hydrogen production, biological hydrogen production, and water electrolysis hydrogen production. Hydrogen production by chemical reforming [4,5] mainly involves high-temperature reforming or partial oxidation reforming of organic matter and fossil fuels, such as coal, natural gas, or petroleum by chemical methods. For example, methane, the main component in natural gas, is decomposed into H_2_, CO_2_, and CO. This kind of hydrogen production technology is quite mature. This route accounts for more than 85% of the current industrial methods, and its hydrogen production rate is 70–90%. The hydrogen prepared by this method belongs to grey hydrogen and blue hydrogen, and blue hydrogen reduces the carbon emission in grey hydrogen through carbon capture technology (CCS). However, the carbon recovery cost in the manufacturing process of blue hydrogen is very expensive, which may increase the fuel consumption by 10% compared with grey hydrogen. The biological hydrogen production method [6,7] uses organic wastes as raw materials to produce hydrogen through photosynthesis or bacterial fermentation. The key point is to cultivate high-efficiency and high-selectivity biological strains, but there are still many problems to be investigated in hydrogen production mechanism, such as strain cultivation, and bacterial metabolic pathway. Water electrolysis hydrogen production [8,9,10] is a completely clean way of hydrogen production, which can be used for peak shaving and energy storage of power stations—that is, the surplus electric energy of power stations is used for hydrogen production by electrolysis of water during the low power consumption period. This kind of technology has been relatively mature internationally, and some domestic hydropower stations and photovoltaic power stations have also applied this technology. The hydrogen obtained by this method possesses high purity and belongs to a class of green hydrogen. Green hydrogen is prepared by electrolytic water with renewable electricity, which not only effectively reduces the carbon emissions in the preparation process but also uses renewable raw materials, which is in line with sustainable development. However, the energy consumption of this method is high, and the on-site hydrogen production at the power station is still limited; the cost is also relatively high, with hydrogen production accounting for about 10%.

According to the prediction of the China Hydrogen Energy Alliance Research Institute, the proportion of hydrogen production from electrolytic water from renewable energy in China will reach 15%, 45%, and 70% in 2030, 2040, and 2050, respectively. With the discovery of renewable energy sources, such as photovoltaic and wind power, and the development of renewable technology, the production cost of electric energy conversion is decreasing, which provides a favorable opportunity for the development and application of hydrogen production by electrolysis of water. With the increasing energy demand, the technology of hydrogen production by electrolysis of water has undergone significant developments. Electrolytic water reaction is mainly composed of two half-reactions: cathodic hydrogen evolution reaction (HER) and anodic oxygen evolution reaction (OER). Due to the slow reaction kinetics, its energy conversion efficiency is low. Generally, noble metal catalysts (such as Pt, Ru, Ir, etc.) are needed to improve the HER/OER reaction kinetics of electrolyzed water to improve the energy conversion efficiency of the system [11,12,13,14]. However, due to the scarcity of resources and high use cost, the application of precious metal-based catalysts in large-scale hydrogen production by electrolysis of water is still limited. Based on this, the current research is focused on developing transition metal-based HER/OER electrocatalysts with high activity, high stability, and low cost. Among many transition metal-based materials, transition metal catalysts based on Ni, Co, and Mo are widely regarded by researchers because of their changeable composition and structure, abundant resources, low cost, and high catalytic activity and stability [15,16,17,18,19,20].

At present, the mainstream hydrogen production technologies include alkaline water electrolysis AWE [21,22], proton exchange membrane water electrolysis PEM [23,24], and solid oxide water electrolysis SOE [25,26], and their corresponding advantages and disadvantages are shown in Table 1. With the continuous development of electrolytic water technology, the efficiency of electrolytic hydrogen production is gradually improving and the energy consumption is decreasing. The decreased energy consumption mainly depends on optimization to improve the electrocatalytic activity of the catalyst. When selecting the catalyst, the following points should be considered: (1) Catalysts should possess high catalytic activity, long service life, and the ability to inhibit catalyst poisoning and complete the electrolytic water reaction. (2) Catalysts should possess high electrical conductivity, which promotes the charge transfer at the interface between the electrode and the electrolyte, thus improving the electrolysis efficiency. (3) Catalysts should possess a large catalytic activity area to promote more reactions. (4) The constituent elements of catalysts are earth-abundant with low cost of large-scale application. (5) Catalysts should possess good electrochemical stability, preventing them from falling off in an acid-based solution or from being poisoned or losing their activity directly because of other impurities and intermediate products of the reaction. Generally, precious metals and their alloy oxides have the best electrocatalytic activity and chemical stability due to their rich empty d orbitals, but their output is scarce and expensive, and the cost of large-scale application is high. Therefore, reducing the loading of precious metals and developing catalysts with low cost, high electrocatalytic activity (with empty d orbitals or not filled with d electrons) and high stability are the keys to improving the hydrogen production efficiency.

The design and development of high-performance catalysts can be regulated by the following two approaches: First, designing new nanomaterials /electrode structures, developing catalytic materials/electrodes with various morphologies/structures, improving the specific surface area and conductivity of catalytic electrodes, constructing open-pore structures, improving the exposure of active sites, and promoting the transport of substances will improve the electrocatalytic activity of HER/OER. This includes the preparation of heterostructure catalysts, rearranging electrons on heterostructure interfaces to modify the properties of active sites, and using the synergy of different active sites to promote the reaction kinetics [27]. Second, the intrinsic activity of electrocatalysts is regulated, and the electronic structure is optimized efficiently by strategies such as heteroatom doping, defect engineering, valence state regulation, and interface engineering, and the adsorption energy of intermediate species in the reaction is improved by regulating the electronic structure of active sites, thus improving the intrinsic activity of the catalysts. At present, there are various limitations to a single optimization method. Combining the advantages of strategies such as the optimization of nanomorphology and microstructure of materials with the regulation of intrinsic defects and compositing with metal or nonmetal carbon materials can effectively improve the catalytic activity of materials and reduce the application cost of materials.

## 2. Brief Introduction of Hydrogen Production Technologies

### 2.1. Alkaline Water Electrolysis Hydrogen Production

**Figure 1:**
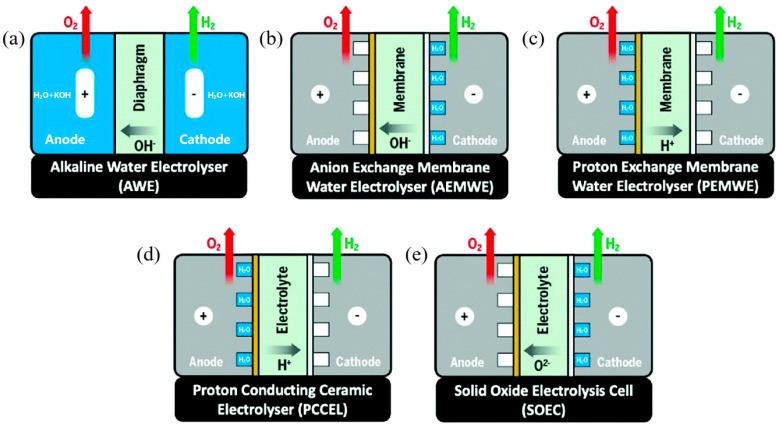
Schematic principle diagram of the five main types of electrolytic water hydrogen production (**a**) AWE, (**b**) AEM (**c**) PEM, (**d**) PCC, (**e**) SOE [34].

Alkaline water electrolysis hydrogen production technology is the earliest industrialized production and the most mature and economical one at present. Its working efficiency is relatively high, generally 42% to 78%. After years of research and development, alkaline water electrolysis technology has made progress in two aspects: first, the efficiency of the electrode has improved, and the operating cost related to electricity consumption has been significantly reduced; second, the operating current density has increased and the investment cost has decreased [28]. The working principle of alkaline water electrolysis is shown in Figure 1 [29]. The electrolytic cell consists of two electrodes, which are separated by an airtight diaphragm. The electrolyzer has a simple structure, low requirements for raw material quality, long service life, generally reaching 10~20 years, and low cost [30]. The electrolytes used in alkaline electrolytic water technology are mainly alkaline electrolytes, such as KOH and NaOH, with a concentration of 20% to 30%. Among them, the KOH solution has high ionic conductivity and a wide application range. The diaphragm is mainly made of porous materials, such as asbestos, ceramics, and nylon. When the electrolysis temperature is 20~90 °C, water is reduced to produce hydrogen in the cathode, and OH^-^ passes through the diaphragm to reach the anode oxidation to produce oxygen. Although alkaline water electrolysis technology has the characteristics of low cost, long service life, mature technology, and stable operation, there are still many shortcomings in its engineering application, such as low current density, poor dynamic response, diaphragm gas leakage, alkali corrosion, and so on. To solve the above problems, researchers have developed an anion exchange membrane technology AEM, which is expected to become an improved scheme of traditional alkaline electrolysis technology and play a technical and cost advantage in large-scale hydrogen production [31].

To some extent, the AEM technology for producing hydrogen by electrolyzing water with an anion exchange membrane is a technique that combines traditional diaphragm alkaline electrolysis and proton exchange membrane electrolysis, which has the advantages of both alkaline electrolysis and PEM electrolysis [32]. AEM technology diaphragm adopts an anion exchange membrane with pure water or weak alkaline solution as an electrolyte, which can realize the circulation of OH^-^ from cathode to anode. The main challenges faced by AEM technology are the lack of AEM with high conductivity and alkaline corrosion resistance and increased cost due to the use of precious metal catalysts. At the same time, CO_2_ entering the contact film will reduce the membrane resistance and electrode resistance, thus reducing the electrolytic performance. Therefore, the main development directions of AEM electrolyzer in the future are as follows: first, develop an AEM diaphragm with high conductivity, ion selectivity, and long-term alkaline stability; second, reduce the use of noble metal catalysts or develop high-performance catalysts without noble metals; and third, reduce the CO_2_ content in the electrolytic cell to improve its electrolytic performance [33].

**Figure 2:**
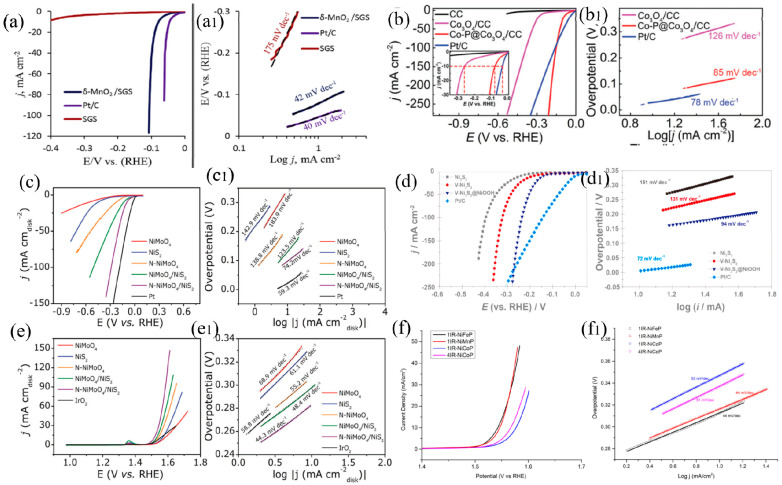
The HER polarization curves and Tafel plots of typical transition metal oxides (**a**–**b1**) [37,38], sulfides (**c**–**d1**) [39,40], phosphide [41] (**f**,**f1**), and OER polarization curves and Tafel plots of transition metal sulfides (**e**,**e1**) [40].

When choosing a catalyst, it is necessary to consider whether it has good electrochemical inertia, electrical conductivity, large specific surface area, catalytic activity, mechanical stability, good thermal conductivity, and strong chemical stability. Precious-based catalysts possess basically all the characteristics needed for high-performance catalysts, but their costs for application and preparation are high, and their scarce reserves and expensive prices greatly limit their wide-range use. In the industrial production of hydrogen from alkaline water electrolysis, the precious metal PtRu catalyst is used in AEM hydrogen production technology, and the widely used catalysts are mainly transition metal catalysts. Transition metals (TMs) have good catalytic activity, low price, and abundant crustal reserves, and are an ideal material to replace precious-based catalysts. TMs catalysts mainly include the following species, such as TMs and their alloys [34,35,36], TM oxides [37,38], TM sulfides [39,40], and TM phosphides [41,42], all of which have excellent hydrogen evolution reaction (HER) and oxygen evolution reaction (OER) activity and stability. After continuous optimization, the performance of some of them can now be comparable to that of precious-based catalysts, which have a very broad application prospect, as shown in Figure 2. The corresponding performance of the typical transition metals-based catalysts is summarized in Table 2.

#### 2.1.1. Transition Metal and Alloy Catalysts

Low-cost transition metals, such as Zr, Nb, Fe, Co, and Ni, possess high electrocatalytic performance in alkaline electrolytes, among which Ni is the most widely investigated and applied because of its good stability in alkaline electrolytes. At the same time, Ni is abundant in the earth’s crust, easy to mine, and relatively low in preparation and use cost. Compared with other metal elements, Ni has a lower oxygen evolution overpotential and a higher oxygen evolution efficiency, so metallic Ni is widely used as an anode material for hydrogen production by electrolysis of water in alkaline electrolytes. The electrocatalytic performance of anode Ni can be optimized by increasing its specific surface area, such as porous Raney Ni anode. On the other hand, the metals on the left side of the transition system in the Periodic Table, such as Fe, Co, and Ni with incomplete or semi-full d orbitals, will have a synergistic catalytic effect on the hydrogen evolution reaction when they form alloy materials with the metals on the right side of the transition system, such as transition metals Zr, W, and Mo with paired d electrons, which will greatly reduce the hydrogen evolution overpotential of the materials. Alloy cathode electrode materials formed by transition metals can be generally divided into three categories [43]: (1) porous Raney Ni alloy materials; (2) Ni-based alloy materials, such as Ni-Mo alloy, Ni-Mo-Fe alloy, and Ni-S alloy; (3) other transition element alloys, such as Fe-R alloy and Fe-Zn-R alloy [44,45]. The typical binary alloys with high catalytic activity are anode alloy electrode materials, such as Ni-Co alloy and Ni-Fe alloy, in which Ni-Co alloy will form a dense inverse spinel NiCo_2_O_4_ oxide film on the electrode surface before oxygen evolution by electrolysis of water, which will greatly reduce the anodic oxygen evolution overpotential due to its high electrocatalytic activity. Additionally, Mn, Fe, and Cu are abundant in the crust and have no pollution effect on the environment. They are also ideal electrode materials. Ternary alloys formed by them and Ni-Co alloys, such as Ni-Co-Fe, Ni-Co-Mn, and Ni-Co-Cu alloys, have improved their electrocatalytic activity to varying degrees. Generally, transition metal and alloy electrodes are prepared by electroplating.

#### 2.1.2. Transition Metal Oxide Catalyst

**Figure 3:**
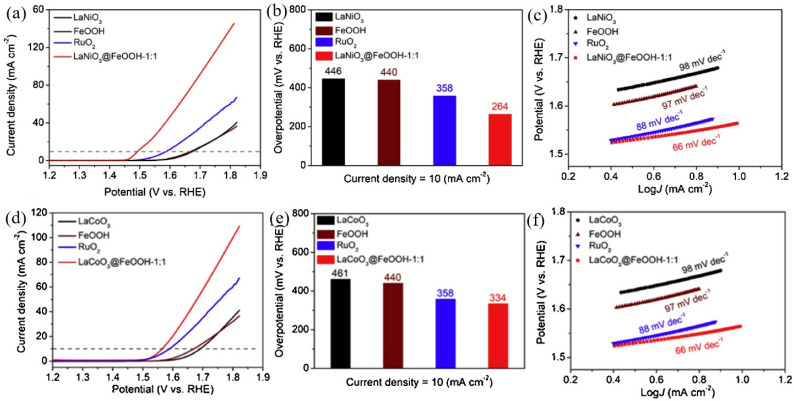
LaNiO_3_, FeOOH, LaNiO_3_@FeOOH−1:1, and RuO_2_ samples: (**a**) LSV curves; (**b**) the corresponding comparison of overpotentials (η); (**c**) the corresponding Tafel plots. LaCoO_3_, FeOOH, LaCoO3@FeOOH−1:1, and RuO_2_ samples: (**d**) LSV curves; (**e**) the corresponding comparison of overpotentials (η); (**f**) the corresponding Tafel plots [47].

The most common perovskite oxides are oxides with ABO_3_ structure formed by lanthanide metal La and transition metals Ni and Co, such as LaNiO_3_ and LaCoO_3_, as shown in Figure 3 [46], which have very high catalytic activity for oxygen evolution. At present, LaNiO_3_ with a pseudo-cubic perovskite structure is the most widely used oxide. LaNiO_3_ is the only metallic compound in perovskite-type oxides, and it is a nonstoichiometric compound. Ni ions exist in bivalent and trivalent forms; there are high-density oxygen holes in the oxides, and there is no forbidden band. The conduction band is formed by the interaction between the d electrons of Ni^3+^ and the p electrons of O^2−^, showing the metal conductivity characteristics [47,48] Therefore, LaNiO_3_ has high metal conductivity and catalytic activity for oxygen evolution. By introducing a small amount of Sr and Mn doping, the catalytic activity of LaNiO_3_ can be greatly improved. Additionally, as shown in Figure 3, LaNiO_3_ and LaCoO_3_ composited with FeOOH possess the highest performance with a low overpotential of 264 mV and 334 mV, respectively; a low Tafel slope of 66 mV dec^−1^, which originates from the high electrical conductivity of LaNiO_3_ and LaCoO_3_; and more active sites due to the existence of FeOOH.

**Figure 4:**
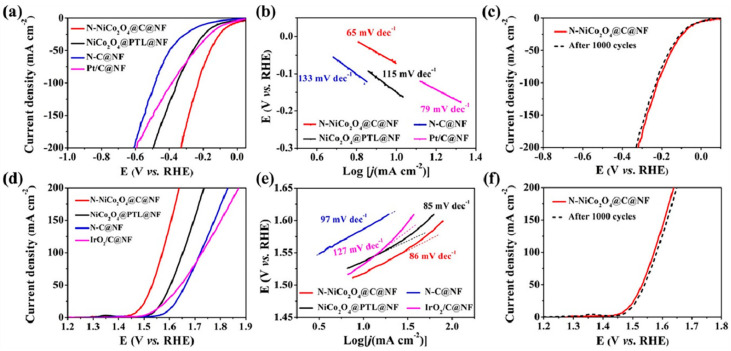
HER and OER performance of N−NiCo_2_O_4_@C, NiCo_2_O_4_@PTL, N−C, and Pt/C catalysts: (**a**) HER LSV curves, (**b**) Tafel plots, and (**c**) LSV curves recorded for N−NiCo_2_O_4_@C before and after 1000 cycles. (**d**) OER LSV curves, (**e**) Tafel plots, and (**f**) LSV curves received for NNiCo_2_O_4_@C before and after 1000 cycles [40].

**Figure 5:**
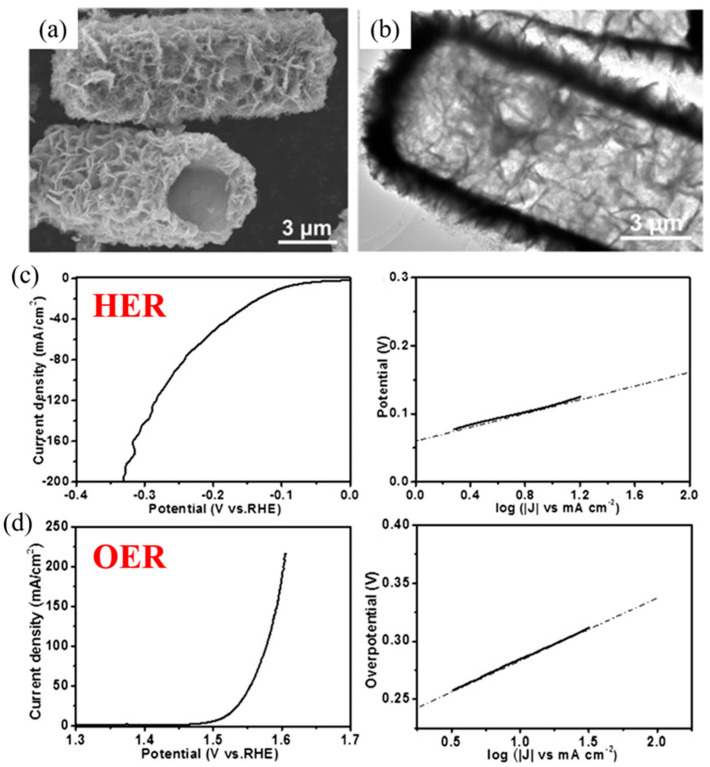
The microstructure and morphology of NiCo_2_O_4_ (**a**,**b**); HER of NiCo_2_O_4_ (**c**) LSV curves and corresponding Tafel plots; OER of NiCo_2_O_4_ (**d**) LSV curves and corresponding Tafel plots [40].

Spinel oxides, typically Co_3_O_4_ and NiCo_2_O_4_, have excellent corrosion resistance in alkaline medium, low cost, and high catalytic activity for oxygen evolution, making them the most promising anode materials for alkaline electrolytic water. Co_3_O_4_ has an inverse spinel structure, which is composed of cobaltous oxide (CoO) and cobaltic oxide (Co_2_O_3_) [49]. Cobalt ions with a valence of +2 occupy tetrahedral gaps, while cobalt ions with a valence of +3 occupy octahedral gaps, which makes the resistivity of Co_3_O_4_ material relatively high (40 Ω m^−1^). By doping and compositing, the electronic interaction among the components in the oxide can produce a synergistic effect, as shown in Figure 2. NiCo_2_O_4_ is a kind of oxide with a spinel structure, in which bivalent Ni ions occupy tetrahedral gaps as A sites, while B sites are trivalent Ni ions occupying oxygen octahedral gaps. This composition with bivalent and trivalent mixed valence can improve its catalytic performance by doping bivalent Ni ions at position A with other transition metal ions [50]. NiCo_2_O_4_ has excellent electrocatalytic performance in OER and HER as shown in Figure 4 and Figure 5 [40]. Its electrical conductivity and chemical stability are optimized by doping, and this makes oxygen adsorbing and desorbing easier. This unique structure gives it high electronic conductivity and chemical stability, as well as good adsorption and desorption performance for oxygen. Generally, methods for preparing transition metal oxide catalysts include solution impregnation (spraying) pyrolysis [51], high-temperature solid-state reaction [52], anodic electrodeposition [53], citric acid sol-gel method [54,55], coprecipitation method [47] and so on.

#### 2.1.3. Transition Metal Sulfide Catalyst

**Figure 6:**
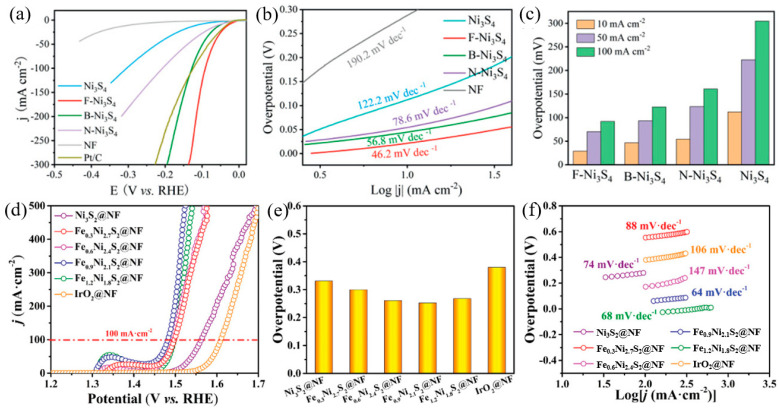
The HER performance of the B−, N−, F−doped Ni_3_S_4_ [58] (**a**) LSV polarization curves, (**b**) Tafel plot, (**c**) overpotential. The OER performance of the Fe−doped Ni_3_S_2_ [59] (**d**) LSV polarization curves, (**e**) overpotential, (**f**) Tafel plot.

Transition metal sulfides (TMSs) have attracted much attention in the domain of electrocatalysis because of their unique structure, composition, and excellent HER activity. The representative TMSs include MoS_2_, Ni_3_S_2_, and WS_2_ [51,56]. Nickel sulfide with different compositions and structures has excellent catalytic activity in HER [57] and OER [58] after doping, as shown in Figure 6. At present, there are different explanations for the reaction mechanism of the S atom in TMSs. Some researchers think that the high electronegativity of the S atom causes it to directly act as the adsorption and analysis site of the H atom. Other researchers believe that sulfide can adjust the electron density by producing S vacancies or constructing an S_δ_-TMn+-H_2_O network to improve the hydrolysis separation process [59]. Plenty of investigations show that the catalytic performance of TMSs can be improved in two ways: one is to increase the exposed number of active sites by increasing the specific surface area of the catalyst, thus improving the catalytic activity; the second is to optimize the catalytic performance by improving the inherent activity of the original active site.

#### 2.1.4. Transition Metal Phosphide Catalyst

**Figure 7:**
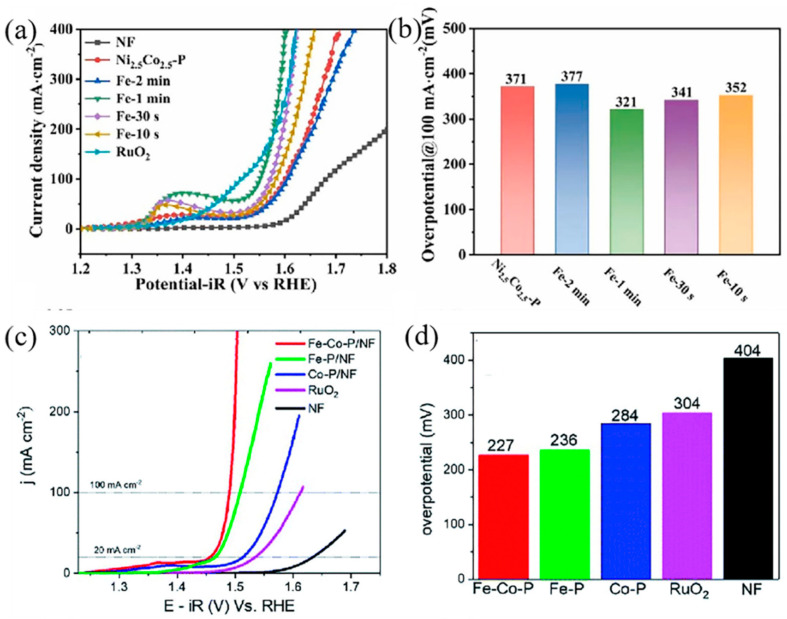
Transition metal phosphides (**a**) the polarization curves for Ni−Co−P after Fe addition (**b**) corresponding Tafel plots [64]; (**c**) OER polarization curves for Multicomponent alloy phosphide, (**d**) corresponding overpotentials [65].

Transition metal phosphides (TMPs) are one of the most widely investigated electrocatalytic materials for hydrogen evolution in HER. P atoms with high electronegativity can extract electrons from neighboring transition metals and capture positively charged protons, so they can be used as active sites to stabilize reaction intermediates [60]. TMPs have stable structure, ceramic and metal characteristics, good thermal and electrical conductivity, and thermodynamic stability, etc., and have always been known as the “quasi-platinum catalyst”. Metal phosphides tend to form more isotropic structures rather than layered structures. Therefore, they can have more coordinative unsaturated surface atoms and are not constrained by the stacking load on the electrode. Furthermore, they have metallic properties, which are beneficial to their application in the electrochemical direction. Typical monometallic TMPs include Ni-P, Co-P, and Fe-P. Compared with monometallic TMPs, bimetallic TMPs, such as Ni-Co-P [61,62,63] and Fe-Co-P [64], can significantly reduce the catalytic overpotential and have more excellent catalytic performance in the field of electrolytic hydrogen production as shown in Figure 7. Brewer-Engel valence bond theory shows that the transition metals with empty or semi-empty d orbitals (d electrons are less than d orbitals) and transition metals with paired d electrons (d electrons are greater than d orbitals) will have a synergistic effect, which is beneficial to the cathodic hydrogen evolution reaction.

### 2.2. Proton Exchange Membrane Water Electrolysis Hydrogen Production

**Figure 8:**
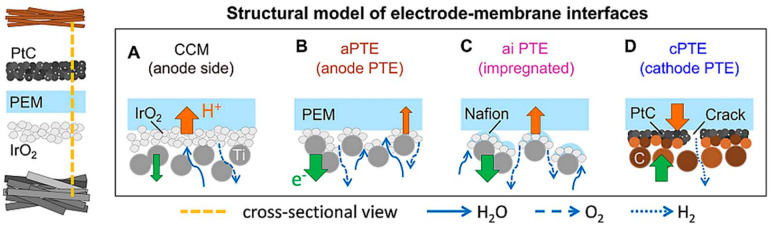
Model of the membrane-electrode interfaces and transport processes in different MEA configurations [68].

Hydrogen production by electrolysis of water with a proton exchange membrane is a technology developed from the 1960s to the 1970s. After a long period of development, its equipment has had a high degree of integration, but there are bottlenecks in many technical aspects. At present, it is mainly imported from developed countries, such as Europe and America. The principle of hydrogen production by PEM is shown in Figure 8. The PEM electrolyzer adopts a bipolar structure, and the electrical connection between cells is carried out by a bipolar plate, which plays an important role in discharging the generated gas [9]. The anode, the cathode, and the membrane group form a membrane electrode assembly. At the anode end, water is catalytically oxidized by the catalyst on the membrane to generate oxygen, electrons, and protons, and the protons generated at the anode are circulated to the cathode end through the membrane and reduced to generate hydrogen. PEM technology uses a perfluorosulfonic acid proton exchange membrane as an electrolytic membrane. Compared with traditional membranes, PEM membrane has the advantages of stable chemical properties, high proton conductivity, nonporous gas isolation, and so on, and can be integrated with electrodes to reduce the extra resistance and power loss caused by the distance between the two electrodes [65]. Therefore, this technology can improve the purity of hydrogen production, and at the same time obtain large current density and rapid response, which is suitable for renewable energy power generation systems with large fluctuations.

However, the investment cost of PEM membrane is high, and almost all the catalysts used depend on platinum-based precious metals and their alloys, as well as precious metal oxides, such as PtO_2_, RuO_2_, and IrO_2_ [66], which greatly increases the use cost. At present, the research on noble metal catalysts mainly focuses on the control of microstructure [67] and composition [68]. Figure 8 shows several methods of membrane electrode assembly in PEM electrolytic cells. When the catalyst layers are directly deposited onto membranes, protonic transport is most likely improved, due to the geometrically shortest protonic pathway for a given PEM thickness, as indicated by the orange arrow. At the anode side, as shown in Figure 8A, only a few titanium fibers directly connected electric contact between the catalyst and membrane layer. When depositing the anode catalysts directly on the titanium diffusion layer, the deposition of large parts of the catalyst layer up to multiple hundreds of micrometers into the pores of the titanium diffusion layer (Figure 8B) is expected. In this case, fewer parts of the catalyst layer are directly in contact with the membrane. Therefore, the reduced protonic interface between the catalyst layer and the PEM leads to an increased ohmic resistance, but the increased interface between the titanium fibers and catalysts makes the electron transport improve, as indicated by the blue arrow. For the anodic porous transport electrodes (aPTE), the carbon-based surface is more planar compared to the titanium substrates. Therefore, one would assume less poorly connected catalyst areas (Figure 8D). For the ai PTE, as shown in Figure 8C, we expected a behavior based on the aPTE and cathode porous transport electrode base cases, but an improvement of the ionic transport and consequently an improved high-frequency resistance. By changing the microstructure of a noble metal catalyst, its electrochemical performance is greatly improved, and the reaction overpotential can be further reduced, or it can be combined with other non-noble metal materials, such as PtCu alloy, to reduce the loading and cost while ensuring its catalytic activity. Furthermore, the typical synthesized electrocatalysts with high electrochemical performance are shown in Table 3. However, compared with AWE technology, PEM technology requires higher water quality [22], which increases not only the production cost but also the supply cost of raw materials. Moreover, due to the imperfect manufacturing process and lack of practical engineering experience, the voltage fluctuates greatly during operation, and the theoretical electrolysis effect cannot be achieved [69]. Although the purity of hydrogen prepared by PEM technology is higher, the preparation cost and service life of membrane and catalysts are difficult to compare with AWE.

Proton conductive ceramic water electrolysis hydrogen production technology PCC uses solid oxide ceramics as electrolytes, which reduces the cost of hydrogen production by PEM [77]. The electrolyte and catalyst used are similar to solid oxide fuel cell SOFC [34], mainly including zirconate, cobaltate, and ferrite perovskite ceramic materials [78]. Among them, the typical perovskite strontium zirconate ceramics show excellent proton conductivity at the temperature of about 700 °C, which is beneficial to the production of high-purity hydrogen at the cathode and can simplify the steam electrolysis device. The main working principle diagram is shown in Figure 1d. The disadvantage of hydrogen production by electrolysis of water with proton conductive ceramics is the overvoltage caused by high ohmic polarization loss at the connection between batteries. Contrary to the tubular battery, the flat battery makes the structure more compact, thus improving hydrogen production efficiency. At present, the main obstacle to the industrial application of hydrogen production technology by electrolytic water with proton conductive ceramics is the long-term stability of electrolyzer in high-temperature and high-humidity environments [79], and there are also problems of electrode aging and deactivation. It works at high temperatures. Ceramic materials are generally used as catalysts, such as perovskite material with proton conduction as an anode and nickel-based ceramic material as a cathode. Ceramic oxide catalysts have excellent high-temperature stability, and their catalytic performance can be optimized by doping and introducing oxygen vacancies.

### 2.3. Solid Oxide Water Electrolysis Hydrogen Production

Solid oxide ceramic is used as an electrolyte in solid oxide electrolytic water technology SOE. Contrary to SOFC, the working temperature is between 500~1000 °C. The high working temperature leads to higher efficiency than AWE and PEM technology, and the highest efficiency can be close to 100% [80]. Hydrogen is generated by water electrolysis under high temperature [81,82]. In the solid oxide electrolyzer, water is converted into water vapor at high temperature, and the current electrolyzes the water molecules adsorbed on the cathode catalytic layer into H^+^ and O^2–^; the free electrons of H^+^ transmitted through the external circuit are reduced to H_2_, and O^2–^ passes through the solid electrolyte layer to reach the anode catalytic layer. At the same time, the lost electrons are converted into oxygen, and the free electrons enter the external circuit [9]. It is required that the electrolyte has high oxygen ion conductivity, so that O^2–^ can pass through the electrolyte layer, and its electronic conductivity is very low to prevent short circuits. The anode and cathode are porous structures, which are beneficial for gas diffusion and constructing the interfaces of three-phase catalytic.

The biggest advantage of SOE technology lies in its high energy conversion efficiency, which can effectively reduce the energy consumption required in the electrolytic procedure [82]. As the PCC technology, its working temperature is higher, and the catalyst used is high-temperature ceramic material. The anode is composed of perovskite or perovskite ceramic composite, and the cathode is made of nickel-based ceramic composite material, so a precious metal catalyst is not needed, and the preparation cost of the catalyst is low [83]. Additionally, SOE technology can also be used for the electrolytic reduction of CO_2_ to realize CO_2_ conversion and emission reduction [82]. Although the catalytic efficiency of SOE technology is increasing with the increase of working temperature, high working temperature also creates some problems, such as difficulty in sealing, and higher requirements for the chemical and mechanical stability of electrodes and catalytic materials in high-temperature and high-humidity environments, which limits the development of SOE technology to some extent. Furthermore, the gas produced by the cathode is a mixture of hydrogen and water vapor, which needs to be further separated and purified, which, in turn, increases the cost compared with conventional liquid water electrolysis. At present, due to the short life of the battery stack, the need for auxiliary components in the process of electrolytic hydrogen production and the high temperature and other safety issues, SOE technology is still in the laboratory research and development stage, and it cannot be commercialized in the short term [8].

After years of research and development, the catalytic performance and chemical stability of electrolyzed water hydrogen production technology have been greatly improved, but there are still some problems with the actual industrial application. First, water electrolysis OER is a slow four-electron transfer process. To drive the reaction, it is necessary to apply a very high overpotential, resulting in high energy consumption. The oxygen evolution performance of the catalyst can be improved by structural optimization. Second, the economic competitiveness of hydrogen production technology by electrocatalytic decomposition of water still needs to be strengthened, and the development of electrocatalysts with low cost and rich resources is still the main research direction in the future. Moreover, the hydrogen evolution reaction in the semi-reaction of electrolytic water is a relatively fast two-electron transfer process, and it is still an effective measure to reduce the energy consumption in the process of electrolytic water by using electrocatalyst to reduce the overpotential of OER and reduce the energy loss in the process of water decomposition. Finally, because acidic or alkaline electrolytes are corrosive, long-term operation will corrode industrial equipment, which further hinders the industrial application of electrolytic water. Therefore, it will be the future development direction to investigate high-performance electrocatalysts in neutral electrolytes. In neutral electrolytes, more materials will be used as suitable electrocatalysts to realize water electrolysis. However, the long-term stability of the catalyst itself is affected by many factors. The researchers found that the current density, electrolyte pH, electrolyte temperature, and the decomposition of the catalytic substrate have a great influence on the long-term stability of the catalytic materials. The optimal parameters could be obtained by comparing the effects of various parameters on the cathodic catalytic hydrogen evolution [84,85,86].

The design of the catalytic electrode structure and the optimization of the catalytic material structure are very important to improve the efficiency of hydrogen production by electrolysis of water. The conventional powder material spraying method uses a binder in electrode preparation, which increases additional resistance, hinders the contact between active sites and electrolyte, hinders the transfer of substances, and leads to the decrease of apparent activity of the catalyst [87]. In situ construction of an integrated electrode on a three-dimensional conductive substrate can effectively improve the conductivity of the electrode, increase the number and utilization rate of active sites, and promote substance transfer. Common three-dimensional electrode substrate materials include carbon-based and metal-based materials, and different substrate materials have different charge transfer capabilities, so their catalytic activities are also different. In addition, different microstructures of catalytic materials have different specific surface areas, which leads to different active sites. Through the microstructure design of the catalyst, the number of active sites exposed on the catalyst surface can be increased, the stability of the catalytic electrode can be improved, and the charge transfer, electrolyte diffusion, and timely release of H_2_ can be improved. By combining the control of different electrode substrates and nanostructures, the catalytic limit of electrodes can be broken, and the material activity and electrolytic efficiency can be effectively improved [88,89]. Therefore, it is necessary to consider a multi-scale design strategy and explore the mechanism of various factors on catalyst activity and stability. For the practical application process of the catalysts, the catalytic activity, and chemical and mechanical stability of the catalytic material need to be considered to ensure that the catalytic electrode material has high catalytic efficiency and service life. To realize large-scale commercial applications, the catalyst’s preparation and use costs need to be thoroughly analyzed. Noble metal catalysts are essential for some noble metals in special catalytic systems due to their excellent catalytic activity and stability. Based on this, it is necessary to consider how to reduce the usage of noble metals on the premise of ensuring the catalytic activity of materials and recycling the subsequent noble metals.

Based on the finding of this study, the future research directions of hydrogen production by water electrolysis mainly include the following: (1) considering the high catalytic activity and electrochemical stability of the noble metal catalyst, especially its excellent stability in acidic electrolysis system, to develop an economical, efficient, and stable electrocatalyst composed of noble metal and base metal [90]; (2) based on the high catalytic activity of the obtained catalyst, further improving the structural stability of the catalyst and preventing large deformation during the reaction; (3) designing an electrolyzer with zero gap structure between anode and cathode to improve the current density and optimize electrolysis efficiency.

## 3. Conclusions

Hydrogen production technology by water electrolysis has the advantages of environmental friendliness, high purity of hydrogen production, and simple preparation process, which has broad development potential under the background of global energy shortage and successive transformation of high-carbon energy. At present, the main technologies for hydrogen production by electrolysis of water can be divided into three categories, including alkaline water electrolysis, proton exchange membrane water electrolysis, and solid oxide water electrolysis. The key to the development of hydrogen production technology by electrolysis of water lies in the selection of catalysts and the optimization of their performance. Optimizing the existing catalyst systems and developing high-performance catalysts, reducing the use cost, and improving the stability of catalysts are of great significance for improving the efficiency of OER and HER, reducing the overpotential of electrodes, and reducing energy consumption. Precious metals, precious metal alloys, and oxides are still the main catalysts with the best performance, but the use cost of precious metal catalysts is high, so it is very important to develop high-performance and low-cost catalysts. Transition metal catalysts are widely used because of their low preparation cost and many optimized electrocatalytic properties comparable to those of precious metal catalysts. Catalysts can be optimized using alloying, element doping, multi-structure compounding, and micromorphology regulation, but the single method of optimization has limitations, and the combination of multi-scale methods can realize the diversified promotion of catalysts from composition and structure to performance. With the increasing demand for clean energy, the technology of hydrogen production from electrolyzed water will be further developed and is expected to boost energy transformation and environmental optimization.

## Figures and Tables

**Table 1:** Type of water-electrolysis hydrogen and its characteristic.

Technologies	Diaphragm	Catalyst		Electrolyte	*T*/°C	Efficiency/%	Advantage	Disadvantage
		Anode	Cathode					
AWE	Porous materials	Ni, Co, Fe, LaCoO_3_, LaNiO_3_, NiCo_2_O_4_	Ni alloy, NiMoO_4_	Alkaline water	20~90	59~70	Low cost, long service life, mature technology	Electrode corrosion, poor dynamic performance
	AEM	Ni-based materials	Ni, NiFe, NiFe_2_O_4_, PtRu/C	Pure or alkaline water	20~200	60~78	Has the advantages of alkaline electrolysis and PEM electrolysis	Low OH^−^ conductivity and poor high-temperature stability
PEM	Perfluorosulfonic acid membrane	RuO_2_, IrO_2_, Ir_1−*x*_Ru*_x_*O_2_	Pt/C, MoS_2_	Polymer, acidic electrolyte	20~200	65~82	Compact design, high responsiveness	High-cost, precious-metal catalyst
	PCC	Perovskite ceramic	Ni ceramic	Ceramic	500~1000	Up to 100%	Low cost, low energy demand, and high electrochemical reaction rate	High cost, poor mechanical stability of ceramics, difficult sealing; easy to cause hydrogen leakage
SOE	ceramic	La*_x_*Sr_1−*x*_MnO_3_, LSM-YSZ	Ni-YSZ, Ni-based ceramic, perovskite	Vapor, ceramic (oxygen ion conductor)	500~1000 °C	Up to 100%		

**Table 2:** Electrochemical properties of typical transition metal-based electrocatalysts.

Transition Metal-Based Electrocatalysts	Catalyst	Overpotential/mV (10 mA cm^−2^)	Tafel Slope/mV dec^−1^	Rct/Ω
alloys	Fe_3_Co_7_@PCNs for HER [35]	220	65.5	47
	Fe_3_Co_7_@PCNs for HER [35]	260	53.16	16
	B-Ti_2_Cu_3_ for HER [36]	155	103.89	14.2
	Mo-Ti_2_Cu_3_ for HER [36]	133	97.37	_
oxides	CoP@Co_3_O_4_@CC for HER [37]	73	85	70
	δ-MnO_2_/SGS for HER [38]	80	42	140
sulfides	V-Ni_3_S_2_/NiOOH for HER [39]	154	94	5.1
	N-NiMoO_4_/NiS_2_ for HER [40]	99	74.2	_
	N-NiMoO_4_/NiS_2_ for OER [40]	283	44.3	_
phosphides	NiFeP for OER [41]	313	44	4.3
	V-CoP/Ni_2_P/NF for HER [42]	20	54.2	2.6

**Table 3:** Electrochemical properties of typical electrocatalysts for PEM electrolyzer.

Electrocatalysts for PEM Electrolyzer	Catalyst	Overpotential/mV (10 mA cm^−2^)	Tafel Slope/mV dec^−1^	Rct/Ω
oxides	Ir_0.6_Mn_0.4_O_x_ for OER [70]	212	40	5.2
	Ir/ATO 70% for OER [71]	256	-	-
sulfides	GDL/(CNTs+FeMoS) for HER [72]	180	57	-
	FeS_2_ for HER [73]	870 mV at 1 A cm^−2^	-	-
phosphides	Ni_78_P_22_ for HER [74]	105	38	1.16
	Ni_71.5_Mo_26.5_P_2_ for HER [75]	28	29	
	20% FeP/CB for HER [76]	51	101	-
